# Patient-Reported Barriers to Healthcare Access Among Patients with Gastrointestinal Cancer: Insights from 45,000 Participants in the All of Us Research Program

**DOI:** 10.21203/rs.3.rs-6855375/v1

**Published:** 2025-07-29

**Authors:** Samuel D. Butensky, Kurt S. Schultz, Elizabeth L. Godfrey, Jihoon Kim, Caroline H. Johnson, Ira Leeds, Sajid A. Khan

**Affiliations:** Yale New Haven Hospital; Yale New Haven Hospital; Yale New Haven Hospital; Yale University; Yale University; Yale New Haven Hospital; Yale New Haven Hospital

**Keywords:** Healthcare Access, Gastrointestinal, Cancer, All of Us

## Abstract

**Background:**

Gastrointestinal (GI) cancer patients often face care delays and cost-related unmet needs, increasing the probability for treatment nonadherence and adverse outcomes. The extent of these barriers within the first three months of diagnosis remains unclear. We aimed to identify early barriers to care for targeted interventions.

**Methods:**

A retrospective analysis using the *All of Us* database included patients with esophageal, stomach, small intestine, pancreatic, hepatocellular, biliary, colorectal, or anal cancer. Patients were stratified into two cohorts based on survey completion. Reasons for delays in care and cost-related unmet needs were included as dependent variables. Propensity score matching (PSM) and logistic regression evaluated the impact of time from diagnosis.

**Results:**

Among 45,061 GI cancer patients, 89.4% were underrepresented in biomedical research. Patients surveyed within three months of diagnosis had higher rates of delays in care (16.9% vs. 14.0%, p < 0.001), driven by affordability, childcare, and transportation (all p < 0.001). Overall cost-related unmet needs did not differ significantly (< 3 months 20.9% vs. >3 months 19.7%, p = 0.204), but differences in unmet prescription and alternative therapy needs persisted. After PSM, early-diagnosis patients had no differences in delays in care but were more likely to report cost-saving behaviors such as using lower-cost prescriptions (OR 1.28, 95% CI 1.05–1.54) and alternative therapies (OR 1.48, 95% CI 1.08–2.01) to save money.

**Conclusion:**

Cost-related unmet needs exist in the first three months after GI cancer diagnosis. This study underscores the importance of addressing social determinants of health early in cancer care.

## Introduction

Cancer patients face significant challenges in accessing healthcare, including affording medications, arranging transportation to appointments, securing time off work, and managing caregiving or childcare responsibilities. Cancer care can also create substantial financial strain, with patients citing cancer as the most common reason to file for bankruptcy due to medical expenses.^1–3^ Furthermore, cancer patients are more likely to report delays in care compared to adults without a cancer history, with approximately 476,000 patients in 2018 experiencing delays.^4^ Ultimately, patients who experience financial hardship and delays in care during cancer treatment have been shown to be at higher risk for treatment nonadherence, poor quality of life, and worse survival.^5–7^

Prior studies utilizing data from the National Health Interview Survey (NHIS) have highlighted these disparities in care access. Analysis of NHIS data has demonstrated a U-shaped pattern in cost-related delayed or forgone care, with early cancer patients—those surveyed within the first year of diagnosis—and long-term cancer patients (10 + years post-diagnosis) reporting the highest rates of financial barriers to care.^8^ However, the patient population captured by NHIS may not be fully representative of the diverse U.S. population, as the cohort has historically been predominantly female, white, and non-Hispanic.^4,8^

Recognizing the need for more representative research, the All of Us Research Program was developed with the goal of building a diverse cohort, prioritizing the recruitment of populations underrepresented in biomedical research (UBR). As part of its data collection efforts, All of Us developed the Healthcare Access and Utilization (HAU) survey, which was modeled after the NHIS survey to assess barriers to care in a more diverse and representative population.^9^ This is especially critical in the context of gastrointestinal (GI) cancers, which often requires multimodal therapy and potentially results in greater out-of-pocket costs, work disruption, and care fragmentation.

Additionally, GI cancer patients’ care may disrupt their ability to afford other aspects of their medical needs, such as prescription medications, resulting in cost-related unmet needs. Given that GI cancer represents one-quarter of all cancer diagnoses worldwide, there is a societal need to understand how delays in care and cost-related unmet needs most impact these patients.^10^ Our study aims to assess barriers to care among GI cancer patients using the prospective survey data from the All of Us database relative to a cancer diagnosis. We hypothesize that patients within three months of diagnosis experience more delays in care and cost-related unmet needs than those diagnosed later.

## Methods

### Data Source and Study Sample

We conducted a retrospective analysis of prospectively collected data from the All of Us Research Program, a nationwide initiative designed to create a diverse health database of over one million United States residents.^11^ The All of Us database has been described in detail.^12^ We used the Controlled Tier Dataset v8, released on October 1st, 2023, encompassing 633,540 patients.

We included individuals aged ≥ 18 years old with a diagnosis of esophageal, stomach, small intestine, pancreas, hepatocellular, biliary, colon, rectum, or anal cancer at any time, defined by SNOMED codes (Supplemental Figure S1). Patients who did not complete the Basics and Healthcare Access and Utilization surveys were excluded. The study adhered to the Strengthening the Reporting of Observational Studies in Epidemiology (STROBE) reporting guideline. As the All of Us database is deidentified, this research was not considered human subjects research and thus was not subject to review under Yale University Institutional Review Board guidelines.

### Survey Responses

The Healthcare Access and Utilization survey is a 57-question survey derived from the National Health Interview Survey (NHIS) that asks questions about access and use of health care.^13^ The survey was subdivided into two categories based on theme: delays in care and cost-related unmet needs. Individual reasons for reporting delays in care and cost-related unmet needs were included as dependent variables in logistic regression. A binary variable was also created to capture any delay in care and any issue affording care: patients who reported ≥ 1 reason for a delay in care or issue affording care were marked “Yes” as having a delay in care or issue affording care, respectively.

### Covariates

Demographic information was obtained from the Basics Survey. Deprivation index was obtained for each person from the “Zip Code Socioeconomic Status Data” concept set within All of Us; deprivation index is an All of Us population-weighted average of the index for the census tracts covered by the 3-digit ZIP code tabulation area (ZCTA). UBR was defined by race, ethnicity, age, household income, disability, education, gender identity, sex at birth, and sexual orientation.^14^ Charlson-Deyo comorbidities were obtained from the electronic medical record using the SNOMED codes (Supplemental Table S1). Race, gender, ethnicity, education, and employment are self-described. Patients with missing data for date of birth, gender, race, or ethnicity were excluded. Responses of “I prefer not to answer,” “None of these,” and “PMI: Skip,” for the demographic and socioeconomic questions were treated as “Prefer not to answer.”

### Justification for 3-Month Cutoff

A binary variable called “Time from Diagnosis to Survey Completion” was created to separate the cohort into patients who answered the survey < 3 months from their cancer diagnosis, and those who completed the survey ≥ 3 months from their diagnosis. While delays in care can occur throughout the first two years post-diagnosis, this interval is too broad to guide timely, targeted interventions. Prior work by Shankaran et al. in the metastatic colorectal cancer population showed that 25% of patients in the first three months after their diagnosis have major financial hardship, and this financial hardship negatively impacts their health-related quality of life.^15^ Based on this literature and institutional experience, the first 90 days post-diagnosis represent a particularly vulnerable period marked by intense decision-making, care coordination, and potential disruption to employment and insurance coverage. Using this time frame allows for more actionable insights into early care disruptions and cost-related unmet needs.

### Statistical Analysis

The patient population was summarized with descriptive statistics. Chi square and Student’s T-Test were used to compare demographic variables. Survey responses were stratified by time from diagnosis to survey completion and statistically compared using Chi square analysis. Propensity score matching (PSM) was performed to match these two cohorts using nearest neighbor methodology in a 2:1 ratio for the following covariates: age, race, ethnicity, gender, comorbidities, deprivation index, income, education, employment, and healthcare insurance. These covariates were chosen due to their relevance to the causal pathway and/or potential for confounding. Standard mean differences were checked before and after matching to ensure balance. Statistical significance was set at an α of 0.05, with a 2-sided *P* value of < 0.05 indicating significance. All analyses were conducted using R in the secure All of Us researcher workbench (workspace: “Gastrointestinal Cancer – SDOH and HUA CT V8”).

### Sensitivity Analysis

To assess the robustness of our findings, two additional analyses evaluated a 6-month and 1-year cutoff of time from diagnosis to survey completion. PSM was used to match these cohorts in similar fashion to the main analysis (Supplemental Figure S2).

## Results

**Figure 1:**
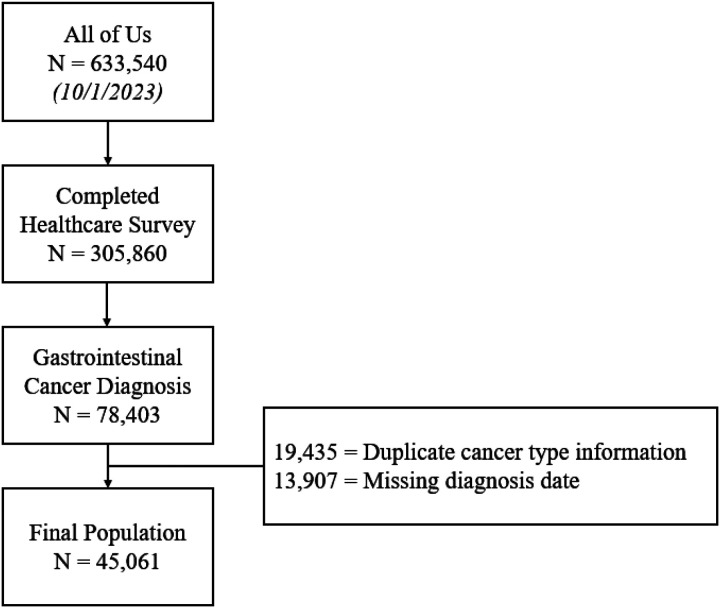
Patient selection criteria for evaluating access to care in patients with gastrointestinal cancer within the All of Us research database

A total of 45,061 patients with a prior diagnosis of gastrointestinal tract cancer met our inclusion criteria (Fig. 1). Most patients were white (70.0%), non-Hispanic (86.2%), and female (54.2%) (Table 1). The majority of patients earned less than $100,000 (54.9%), and the most common cancer diagnosis was colorectal cancer (67.8%). 89.4% were UBR; of those who were UBR, 77% were > 65 years old, 25% had at least one disability; 36% earned < $50,000, and 34% were non-white. There were significant differences between diagnosis to survey cohorts in all covariates except gender (p = 0.36820). 9% of patients who completed the survey within 3 months had cost-related unmet needs, compared to 19.7% of patients who completed the survey ≥ 3 months after their diagnosis (p = 0.204). The number of reasons for delays and cost-related unmet needs decreased over time (Supplemental Figure S3).

### Delays in Care

**Figure 2:**
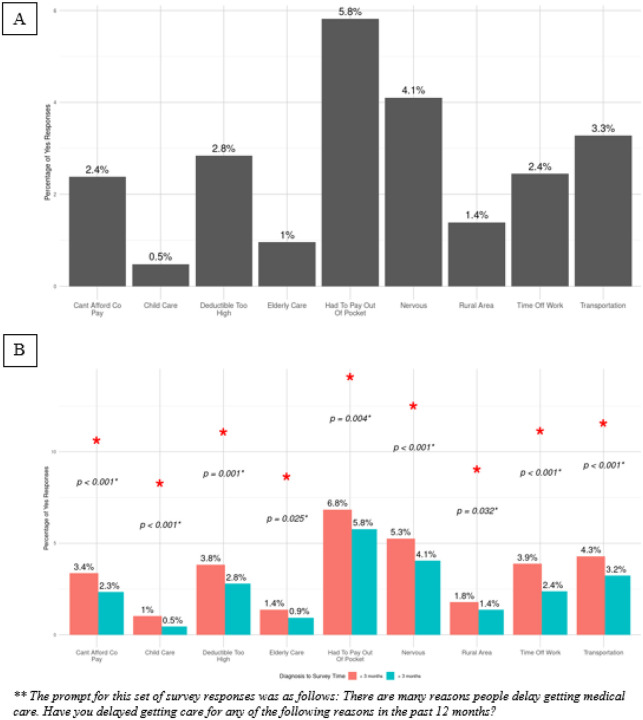
Reasons for delays in care in A) patients with gastrointestinal cancer then B) stratified by time from cancer diagnosis to survey completion.

14.1% of all respondents reported delays in cancer care, with 16.9% of patients < 3 months from their diagnosis having delays in care as compared to 14.0% in the other cohort (p < 0.001) (Table 1). 5.8% of patients delayed care because they had to pay out of pocket for their visit. 4.1% of patients delayed care because they were nervous, and 3.3% of patients delayed care due to issues with transportation (Fig. 2A). When stratified by diagnosis to survey completion time (Fig. 2B), there were significant differences in every category. When compared to patients greater than 3 months after their diagnosis, more patients within 3 months of their diagnosis delayed care because of issues affording their copay (3.4% vs. 2.3%, p < 0.001), childcare (1% vs. 0.5%, p < 0.001), elderly care (1.4% vs. 0.9%, p = 0.025), issues getting time off work (3.9% vs. 2.4%, p < 0.001), and difficulty finding transportation (4.3% vs. 3.2%, p < 0.001). After PSM, there were no significant differences in these two cohorts (Fig. 4A).

### Cost-related Unmet Needs

**Figure 3:**
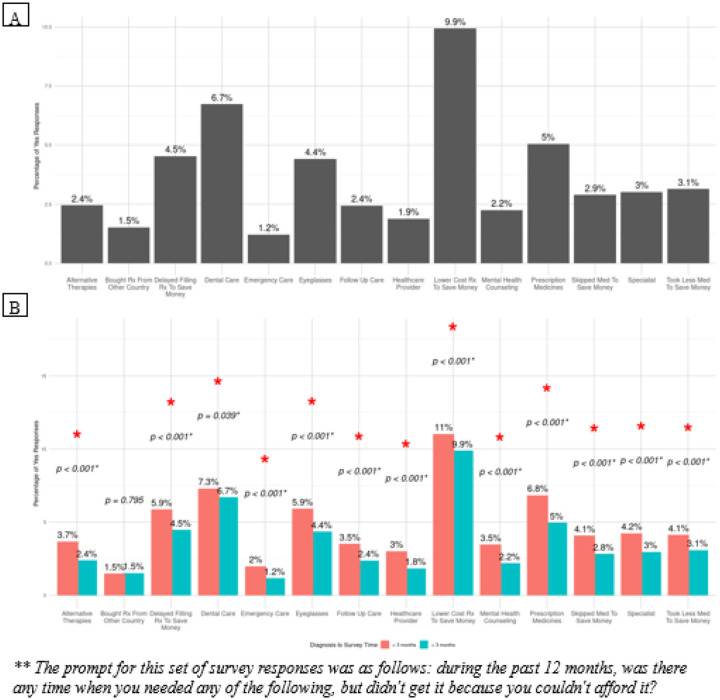
Reasons for cost-related unmet needs in A) patients with gastrointestinal cancer then B) stratified by time from cancer diagnosis to survey completion.

**Figure 4:**
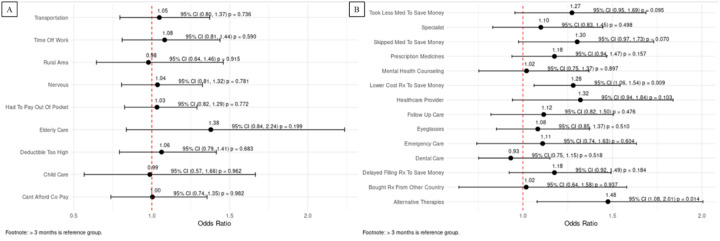
Binomial logistic regression evaluating the effect of diagnosis from survey time on A) delays in care and B) cost-related unmet needs in a propensity score matched cohort

19.7% of all patients reported cost-related unmet needs, with 20.9% of patients < 3 months from their diagnosis having cost-related unmet needs compared to 19.7% in the other cohort (p = 0.204). 9.9% of patients asked their doctor for a lower cost medication to save money. 6.7% of patients needed dental care, 5% needed prescription medications, and 4.4% needed eyeglasses but were unable to obtain them due to financial constraints (Fig. 3A). 3.1% of patients took less medication to save money. When stratified by diagnosis to survey completion time (Fig. 3B), all categories, except buying a prescription from another country to save money, had significant differences. As compared to patients who completed the survey more than 3 months after their cancer diagnosis, more patients within three months of their diagnosis took a lower cost prescription to save money (10.8% vs. 9.8%, p < 0.001), needed dental care but could not afford it (7.5% vs. 6.6%), needed prescription medication but could not afford it (6.5% vs. 4.8%, p < 0.001), and used alternative therapies to save money (3.0% vs. 2.4%, p < 0.001). After PSM, two significant differences remained (Fig. 4B). Patients within 3 months of their diagnosis had increased odds of taking a lower cost prescription (OR 1.28, [95% CI: 1.05–1.54]) and trying alternative therapies (OR 1.48, [95% CI: 1.08–2.01]) to save money, as compared to patients more than three months after their cancer diagnosis.

### Sensitivity Analysis

For the binomial logistic regression on delays of care, there remained no significant differences closer to patients’ cancer diagnosis as compared to later on at both the 6-month and 1-year threshold (Supplemental Figure S4). With regard to cost-related unmet needs, taking a lower cost prescription to save money remained persistently higher closer to the time of diagnosis at both the 6-month and 1-year threshold (Supplemental Figure S5). Trying alternative therapies to save money was not robust after a sensitivity analysis. All other reasons for cost-related unmet needs remained non-significant in the sensitivity analysis, except that patients at the 6-month cutoff were more likely to need prescription medications but not be able to afford them (OR 1.23 [95% CI: 1.03–1.46]) (Supplemental Figure S5B); this finding was not significant at the 3-month and 1-year cut points.

## Discussion

This study examines challenges related to healthcare access and affordability among a national cohort of patients with GI cancer. Overall, 14.1% reported care delays, with patients in their first three months of diagnosis more likely to report problems related to out-of-pocket costs, transportation, childcare, and time off work. Similarly, 19.7% reported cost-related unmet needs, with early respondents more often reporting medication non-adherence, unmet dental or prescription needs, and use of alternative therapies. These results suggest that the acute time period after a cancer diagnosis is a sensitive period that may impact their cancer care as well as their ability to access other aspects of their healthcare.

While it has been well-established that living with cancer can increase cardiovascular diseases, suicide and psychiatric symptoms and disorders, the diagnosis itself can also be highly stressful.^16–18^ Lu et al. showed that compared to the two years before a cancer diagnosis, patients who were diagnosed with cancer had a peak in depression, anxiety, substance abuse, and stress reactions by the first week of their diagnosis.^19^ While the number of disorders decreased rapidly, they still remained elevated 10 years after diagnosis. This peak in emotional stress after a cancer diagnosis is compounded by the logistical maze patients must navigate to secure treatment while keeping the rest of their lives moving forward.

Our study reflects these challenges, with patients within the first three months of their diagnosis experiencing increased delays in care and cost-related unmet needs than patients more than 3 months out. It might also explain why patients within the first three months of their cancer diagnosis were more likely to use lower cost prescriptions and seek alternative therapies, and they associated these behaviors with cost considerations. The use of complementary and alternative medicine (CAM) in cancer care is widespread, with recent estimates indicating that up to 87% of individuals diagnosed with cancer utilize at least one form of alternative therapy following their diagnosis.^20^ Reasons for using CAM vary, including managing cancer and treatment-related symptoms and side effects, enhancing quality of life, and providing a sense of control.^21–25^

With regards to delays in care, the process of delaying treatment is complex. Several studies have shown that traditional risk factors such as age, race, ethnicity, clinical staging, tumor size, treatment facility, and the number of comorbidities contribute to treatment delays.^26–28^ Others have examined the role of social determinants of health (SDOH), including insurance status and geography, in these delays.^29–32^ However, our study utilizes patient-level data to focus on care delays, particularly those based on psychosocial factors. We found that 4.1% of GI cancer patients delayed care due to nervousness, 2.4% postponed care because they could not take time off work, and 0.5% (~ 225 patients) delayed care owing to childcare issues.

The existing literature on the impact of these patient-level factors is limited. Frosch et al. interviewed 22 cancer patients and discovered that psychological distress both contributes to and results from treatment delays.^33^ Conversely, other studies suggest that higher anxiety levels and awareness of cancer symptoms can reduce treatment delays. Regarding childcare, women are especially vulnerable to postponing care due to childcare responsibilities—Gaur et al. found that among patients awaiting ambulatory services, 54.5% of women reported missing or delaying care because of these issues.^34^ Further explanatory studies are essential to clarify the effects of these barriers on delays in care.

In addition to delays in care, prior studies utilizing NHIS or Medical Expenditure Panel Survey (MEPS) have demonstrated significant a tendency to forgo treatment altogether because of cost, resulting in cost-related unmet needs;^2,8,35^ while NHIS gathers data from face-to-face interviews with patients, the MEPS also includes medical providers and employers across the United States. Weaver et al. examined 6,602 cancer survivors using the NHIS and found 10% deferred prescription medication and 11.3% forewent dental care.^8^ Our findings indicate that 2.9% forwent prescription medications and 6.7% forwent dental care. Part of the reason that our results have lower proportions than Weaver et al. is that their study population is not UBR. In turn, given the high proportion of UBR in our research, our findings may offer a more accurate representation of financial delays in care. Additionally, our study focuses specifically on GI cancer patients rather than all cancer patients.

Several actions can be taken in light of our study’s findings. Oncology clinics should consider enhancing access to financial navigation programs to optimize insurance, co-pay assistance, and prescription affordability. Evidence-based complementary therapies could be incorporated into cancer care with insurance coverage. Patient navigators and social workers should proactively address financial toxicity within the first three months after diagnosis. Hospitals should provide subsidized childcare and flexible work accommodation to minimize care delays. Transportation assistance programs, such as ACS’s Road to Recovery, should be expanded. EHR-based psychosocial screenings should result in mental health referrals. Finally, policy advocacy should concentrate on Medicaid expansion, drug pricing reform, and increased federal funding to alleviate financial toxicity in oncology care.

Future studies should incorporate the role of psychosocial risk factors on delays and affordability of care.^36^ All of Us contains an SDOH survey with several psychosocial domains that provide unprecedented patient-level reporting on risk factors that have been poorly studied in the literature. Additionally, we would like to look at the impact of time from treatment or intervention to survey completion on delays and care affordability. These next steps will inform the design of targeted psychosocial interventions to improve access to care and outcomes in patients with cancer.

### Limitations

As a retrospective analysis, this study cannot establish causal relationships between the time from diagnosis to survey completion and healthcare access challenges in GI cancer patients. Response bias remains a limitation, as our cohort was disproportionately female, highly educated, and non-Hispanic white, which may limit generalizability. However, our cohort was 90% UBR, comparable to or exceeding prior All of Us studies.^37,38^ Additionally, insurance type is not available in the *All of Us* database. Lastly, we did not assess cancer-specific delays and costs-related unmet needs, limiting disease-specific insights.

## Conclusion

This study identifies significant financial and access barriers among patients with GI cancers, particularly within the first three months following their cancer diagnosis. Nearly 90% of the cohort was UBR, and despite 97% being insured, cost-related unmet needs persisted. Delays in care were driven by affordability, childcare, and transportation barriers, but these factors were non-significant after PSM. After PSM, patients who completed the survey within 3 months of their cancer diagnosis were more likely to take lower-cost prescriptions and use alternative therapies to save money. While delays were no longer significant after adjustment, affordability challenges remained. Addressing affordability of care is essential for equitable GI cancer care.

## Supplementary Files

This is a list of supplementary files associated with this preprint. Click to download.

- SCCAoUHAUSupplemental.docx

## Figures and Tables

**Table 1:** Clinical and demographic information of patients with gastrointestinal cancer in our study population stratified by time from diagnosis to survey completion.

Variable	Overall	Diagnosis to Survey Time		P Value
		< 3 Months	> 3 Months	
**n**	45061	1959	43102	
**UBR, n (%)**	40271 (89.4)	1664 (84.9)	38607 (89.6)	<0.001
**Delays in Care (Yes), n (%)**	6345 (14.1)	332 (16.9)	6013 (14.0)	<0.001
**Cost-Related Unmet Needs (Yes), n (%)**	8893 (19.7)	409 (20.9)	8484 (19.7)	0.204
**Diagnosis to Survey (Years), n (%)**	6.39 (5.41)	0.11 (0.07)	6.67 (5.36)	<0.001
**Age, mean (sd)**	68.72 (11.61)	62.18 (13.15)	69.02 (11.44)	<0.001
**Gender, n (%)**				0.368
Male	20042 (44.5)	840 (42.9)	19202 (44.6)	
Female	24442 (54.2)	1098 (56.0)	23344 (54.2)	
Non Binary	98 (0.2)	4 (0.2)	94 (0.2)	
None Indicated	479 (1.1)	17 (0.9)	462 (1.1)	
**Ethnicity, n (%)**				<0.001
Hispanic	4764 (10.6)	335 (17.1)	4429 (10.3)	
Non-Hispanic	38843 (86.2)	1568 (80.0)	37275 (86.5)	
Prefer not to answer	1454 (3.2)	56 (2.9)	1398 (3.2)	
**Race, n (%)**				<0.001
White	31533 (70.0)	1195 (61.0)	30338 (70.4)	
Asian	787 (1.7)	56 (2.9)	731 (1.7)	
Black or African American	6270 (13.9)	311 (15.9)	5959 (13.8)	
Other	6471 (14.4)	397 (20.3)	6074 (14.1)	
**Comorbidities, n (%)**				<0.001
< 1 Comorbidity	19006 (42.2)	865 (44.2)	18141 (42.1)	
1–3 Comorbidities	18420 (40.9)	852 (43.5)	17568 (40.8)	
4 + Comorbidities	7635 (16.9)	242 (12.4)	7393 (17.2)	
**Deprivation Index, mean (sd)**	0.31 (0.06)	0.32 (0.07)	0.31 (0.06)	<0.001
**Income, n (%)**				<0.001
150k+	6293 (14.0)	282 (14.4)	6011 (13.9)	
100k–150k	5756 (12.8)	223 (11.4)	5533 (12.8)	
50k–100k	10418 (23.1)	365 (18.6)	10053 (23.3)	
< 50k	14347 (31.8)	650 (33.2)	13697 (31.8)	
Prefer not to answer	8247 (18.3)	439 (22.4)	7808 (18.1)	
**Education, n (%)**				<0.001
Advanced degree	12268 (27.2)	462 (23.6)	11806 (27.4)	
College	10986 (24.4)	479 (24.5)	10507 (24.4)	
High School	18543 (41.2)	833 (42.5)	17710 (41.1)	
Did not graduate high school	2467 (5.5)	151 (7.7)	2316 (5.4)	
Prefer not to answer	797 (1.8)	34 (1.7)	763 (1.8)	
**Employment, n (%)**				<0.001
Employed	14893 (33.1)	775 (39.6)	14118 (32.8)	
Unemployed	29405 (65.3)	1141 (58.2)	28264 (65.6)	
Other	763 (1.7)	43 (2.2)	720 (1.7)	
**Insurance, n (%)**				0.011
No health insurance	708 (1.6)	47 (2.4)	661 (1.5)	
Yes health insurance	43633 (96.8)	1881 (96.0)	41752 (96.9)	
Unknown	720 (1.6)	31 (1.6)	689 (1.6)	
**Cancer, n (%)**				<0.001
Colorectal	30543 (67.8)	1144 (58.4)	29399 (68.2)	
Anus	11827 (26.2)	568 (29.0)	11259 (26.1)	
Biliary	412 (0.9)	48 (2.5)	364 (0.8)	
Esophageal	197 (0.4)	25 (1.3)	172 (0.4)	
Hepatocellular	230 (0.5)	34 (1.7)	196 (0.5)	
Pancreas	571 (1.3)	74 (3.8)	497 (1.2)	
Small Intestine	266 (0.6)	23 (1.2)	243 (0.6)	
Stomach	1015 (2.3)	43 (2.2)	972 (2.3)	

## Data Availability

The All of Us Research Program is a publicly available, de-identified database (https://databrowser.researchallofus.org/). All supporting code used to analyze the data can be found in the All of Us researcher workbench (workspace: “Gastrointestinal Cancer – SDOH and HUA CT V8”).
